# Fresh insights into the pyrimidine metabolism in the trypanosomatids

**DOI:** 10.1186/s13071-018-2660-8

**Published:** 2018-02-08

**Authors:** Kartikeya Tiwari, Vikash Kumar Dubey

**Affiliations:** Department of Biosciences and Bioengineering, Indian Institute of Technology Guwahati, Guwahati, Assam 781039 India

**Keywords:** Trypanosomatids, *Trypanosoma*, *Leishmania*, *Crithidia*, Pyrimidine

## Abstract

The trypanosomatid parasites continue their killing spree resulting in significant annual mortality due to the lack of effective treatments and the prominence of these diseases in poorer countries. These dimorphic parasites thrive unchecked in the host system, outsmarting the immune mechanisms. An understanding of biology of these parasitic forms will help in the management and elimination of these fatal diseases. Investigation of various metabolic pathways in these parasites has shed light in the understanding of the unique biology of the trypansomatids. An understanding of these pathways have helped in tracing the soft targets in the metabolic pathways, which could be used as effective drug targets which would further impact the therupeutic implications. Pyrimidine pathway is a vital metabolic pathway which yields in the formation of pyrimidines, which are then integrated in nucleic acids (DNA and RNA) in sugars (UDP sugars) and lipids (CDP lipids). A wealth of data and information has been generated in the past decades by in-depth analyses of pyrimidine pathway in the trypanosomatid parasites, which can aid in the identification of anomalies between the parasitic and host counterpart which could be further harnessed to develop therapeutic interventions for the treatment of parasitic diseases. This review presents an updated and comprehensive detailing of the pyrimidine metabolism in the trypansomatids, their uniqueness and their distinctions, and its possible outcomes that would aid in the eradication of these parasitic diseases.

## Background

Trypanosomatids are a group of parasites which are the causative agents of deadly diseases such as African sleeping sickness, chagas disease and visceral leishmaniasis, and have been inflicting huge morbidities especially in third world countries [1–3]. Although the past few years has seen mild success in decreasing cases of these deadly parasitic infections, especially African sleeping sickness, but efforts are on the way to improve the current situation. Some new targets in these parasites have been identified and exploited in the past for the broadening of current treatment options. Basic understanding of the metabolic pathways of these parasites is of paramount importance for novel drug/drug target discovery. Pyrimidine pathway has been investigated in great detail in the past decades, which has delineated its regulation in the trypanosomatids [4, 5]. Although little intervention has been sought based on the understanding of the pyrimidine metabolism in the trypansomatids, but large dataset and key differences between the host and parasite would aid in future developments. A range of biochemical, genetic and structural studies have detailed the intricacies of pyrimidine metabolism in the trypanosomatids and the present review aims at analyzing pyrimidine metabolism in the trypansomatids which would help understanding this ancient pathway and scope of drug discovery. Morover, crucial differences and contribution of these metabolic pathways in the growth and infectivity of the parasites is also delineated. Overall, the review gives a snapshot of the current status of pyrimidine metabolism in the trypansomatids.

## Synteny of pyrimidine pathway genes in trypanosomatids

All the genes coding for the *de novo* pyrimidine biosynthetic enzymes are arranged in an operon-like manner suggesting their retrieval in a lateral manner [6]. The clustering of genes encoding the *de novo* pyrimidine biosynthesis enzymes in the trypanosomes was first noted by Aoki et al. [7]. The genes coding for all the six enzymes of *de novo* pyrimidine biosynthesis pathway in the *T. cruzi* genome are arranged in the following order: pyr1-pyr3-pyr6/5-pyr2(ATC)-pyr4, thus reflecting the syntenic arrangement of *de novo* pyrimidine pathway genes in the trypanosomatids. Similar clustering or synteny has also been observed in other related trypanosomatids [5]. This syntenic arrangement of the pathway genes represents an early evolution and common ancestry of trypanosomatids, as many genes of the same pathway are usually found to be clustered in the trypanosomatids. Although *Trypanosoma brucei* exhibited a loss of synteny at the dihydroorotase (*DHOase*) locus [8], a similar clustering of genes was not observed in the case of uracil phosphoribosyltransferase (*UPRT*) gene and other genes coding for the interconversion enzymes of the pyrimidine pathway. This signifies that the observed synteny was specific to the *de novo* pyrimidine pathway enzymes. A hypothetical protein-coding gene and several histone coding genes have also been found to be clustered along with the *de novo* pyrimidine pathway genes in *Leishmania major*, *Leishmania mexicana* and *Leishmania infantum*, but its functional relevance has not been deduced [9].

## Enzymes of the pyrimidine pathway of trypanosomatids

### Carbamoyl phosphate synthetase II

*Trypanosoma cruzi* is the causative agent of a vector borne chronic disease referred to as chagas disease prominent in the Latin America and Mexico and is characterized by the cardiovascular and neurologic manifestations [10]. Kinetoplastids such as *Trypanosoma* and *Leishmania* have well-developed and functional *de novo* and salvage pathway for the biosynthesis of pyrimidines [11–13]. The first three steps in the *de novo* pyrimidine pathway of *T. cruzi* should be catalyzed by independent single enzymes, a notion which has been stemmed from the finding that the open reading frames (ORFs) coding for these enzymes have leader sequences and mRNA splice sites [14, 15]. Also, it was hinted that the enzymes may not interact covalently but may exhibit weak interactions. However, a multimeric enzyme was shown to exist in *T. cruzi* with the help of co-immunoprecipitation and LC-MS-based studies ending speculation. CPSII (first enzyme) co-immunoprecipitated ATCase (second enzyme) and DHOase (third enzyme), thus confirming their molecular interactions. Reverse co-immunoprecipitation experiments further confirmed the non-covalent association of the three enzymes in *T. cruzi* [16]. *Trypanosoma cruzi* closely resembles its host in the organization of first three steps of the *de novo* pyrimidine pathway [16], a finding that may be true in other trypanosomatids where independent existence of enzymes has been proposed. Existence of three independent enzymes has been proposed in *Leishmania donovani*, unlike that of mammalian cells which possess a multifunctional enzyme (CAD) to carry out the first three steps of the *de novo* pyrimidine biosynthesis. Independent activities of the first three enzymes of the *de novo* pyrimidine pathway in *L. donovani* were demonstrated by separating the crude purified extract on Sephadex G-100 column. However, very low activity for carbamoyl phosphate synthetase was observed [17]. There have been similar reports of independent existence of first three enzymes of *de novo* pyrimidine pathway from *Crithidia luciliae*.

Carbamoyl phosphate synthetse II (CPSII) is the first enzyme of the *de novo* pyrimidine biosynthesis pathway, catalyzing the formation of carbamoyl phosphate. Genetic and functional studies of CPSII in the *T. cruzi* and *L. donovani* have shed light on the vital requirement of the first enzyme in the metabolic pathway. Null mutants of CPSII in *T. cruzi* and *L. donovani* had retarded growth phenotype which could be reversed by pyrimidine supplementation. TcCPSII null mutants in the amastigote stage of *T. cruzi* had pronounced retarded growth phenotype, primarily because of their inability to acquire nucleotides from the cellular pool, which are abundant in cellular system. It is noteworthy that the amastigote stage of *T. cruzi* prefers the uptake of pyrimidines and nucleosides over nucleotides, which supports the retarded growth phenotype of TcCPSII null mutants [4, 18–20]. The *de novo* route of pyrimidines is the primary pathway for *T. cruzi* amastigotes, as they are poor uptakers of pyrimidines. Hofer et al. also demonstrated that bloodstream form *Trypanosoma* failed to incorporate [^3^H]-cytosine or [^3^H]-cytidine in their nucleotide pool [19, 21]. Various pyrimidine analogs have been tested for their inhibitory effects on the *T. cruzi* growth. Among the pyrimidine analogs tested 3′-azido-3′-deoxythymidine (zidovudine) displayed significant inhibition at a concentration of 1 μM [22].

Defective growth phenotype of LdCPSII null mutants could be reversed by supplementation of uracil, uridine, deoxyuridine, cytidine, deoxycytidine, orotate and dihydroorotate. LdCPSII null mutants also had reduced infectivity, which could be restored by exogenously expressing LdCPSII. This is in stark contrast with *T. cruzi*, as *L. donovani* has the ability to salvage (transport) nucleotides. Studies of CPSII null mutants of *T. cruzi* and *L. donovani* have established the fact that *L. donovani* has better access to the nucleotide pools, compared to the *T. cruzi* amastigotes which are poor uptakers and thus rely completely on the *de novo* route of pyrimidine synthesis. This supports the notion that the *de novo* pathway in *T. cruzi* could offer potential vulnerable drug targets, while for *L. donovani* targeting a single pathway remains a tedious task. The CPS (LdCPSII) in *L. donovani* carries a glutamine amido transferase domain at the N terminal, along with the catalytic CPS domain separated by the polylinker region, making it structurally distinct even from the prokaryotes which code different polypeptides for these two separate enzymes.

CPSII, being the first enzyme of the *de novo* pyrimidine biosynthesis pathway, is subject to feedback regulation in trypanosomatids which has been verified in *Crithidia fasciculata* CPSII which was inhibited by UTP, UDP and CTP in a feedback manner. Separate existence of CfCPSII has been supported, as the enzyme was separate from the ATCase which is otherwise clubbed together in the mammalian counterpart [23, 24]. Regulation of CPSII in trypanosomatids is different to the mammalian CPSII, as the PRPP does not inhibit CfCPSII but inhibits mammalian CPSII. Similarly, nucleoside diphosphates like UDP and CDP exert more inhibitory effects on CfCPSII, compared to the mammalian CPSII which is inhibited in a feedback manner by UTP. UTP was only able to exert minimal inhibitory effects on ClCPSII activity in *Crithidia luciliae*. This variation in the regulation of the CPSII enzyme of trypanosomatids may represent an early evolution of trypansomatids. Preliminary biochemical studies on CPSII have been carried out in *C. luciliae*. ClCPSII exhibited a *K*_*m*_ of 22.9 μM with glutamine as a substrate. Acivicin, which is also an antitumor compound, is among the few compounds that have been tested for the inhibition of CPSII of trypansomatids. Acivicin, which is a glutamine analog, was able to inhibit CfCPSII by inactivating the L-glutamine dependent activity of CfCPSII with a *K*_i_ of 2 μM [25].

### Aspartate transcarbamoylase (ATCase)

Aspartate transcarbamoylases **(**ATCases) in the trypanosomatids display a variety of distinctness in terms of their structure, which has been exemplified by structural and functional studies. *Trypanosoma* ATCase is unresponsive to CTP levels in the cell as it lacks any regulatory domain, while mammalian ATCase is responsive to CTP levels. It has been reasoned that mammalian ATCase is a part of same polypeptide chain which forms a CPSII domain along with ATCase, thus CPSII being regulated by CTP levels also influences the activity of ATCase while in the case of TcATCase, which associates with the TcCPSII in a non-covalent manner is unaffected by the CTP levels. This is a key distinction between the host and TcATCase. ATCase in trypanosomatids displays variation in oligomeric arrangements, as TcATCase is a homotrimer while LdATCase is a tetramer. Homotrimeric structure of TcATCase has been shown by its crystal structure which diffracted at 2.8 Å and is also supported by gel filtration chromatography and dynamic light scattering studies. In the crystal structure of TcATCase the substrate (carbamoyl phosphate) was bound to loop residues (Cys85-Thr95) which otherwise exhibited a disordered arrangement in the ligand free state [26].

Biochemical investigation of ATCase in trypanosomatids has been done for *Leishmania* and *Crithidia* species. *Leishmania donovani* ATCase (LdATCase) catalyzed reaction proceeded with a *K*_*m*_ of 3.1 × 10^−4^ M (for carbamoyl phosphate) and 7.6 × 10^−3^ M (for aspartate) following a hyperbolic kinetics. ClATCase catalyzed reaction proceeded with a *K*_*m*_ of 28.7 μM (for carbamoyl phosphate) and 2.6 μM (for aspartate), respectively. CfATCase catalyzed reaction proceeded with a *K*_*m*_ of 0.5 mM (for carbamoyl phosphate) and 5 mM (for aspartate), respectively. ClATCase displays higher affinity to its substrates then its *L. donovani* and *C. fasciculata* homologs. A pH optimum of above 9.0 was observed for ATCase of *C. fasciculata*. *Crithidia fasciculata* ATCase (CfATCase) also demonstrated substrate inhibition by aspartate at a concentration above 10 mM. The activity of CfATCase remained unaffected in the presence of pyrimidine ribonucleotides.

N-(Phosphonoacetyl)-L-aspartic acid (PALA) is a specific inhibitor of mammalian ATCase. PALA inhibited LdATCase with a *K*_i_ of 0.5 μM, but it failed to inhibit TcATCase, signifying the fact that TcATCase are more diverse as compared to LdATCase [27].

### Dihydroorotase (DHOase)

Dihydroorotase has been biochemically characterized in *L. donovani*, *C. fasciculata* and *C. luciliae*. LdDHOase catalyzed the forward reaction with a *K*_*m*_ and *V*_*max*_ of 28.1 ± 6.5 μM and 1.2 ± 0.06 μM/s, while the reverse reaction proceeded at a *K*_*m*_ and *V*_*max*_ of 602 ± 79.5 μM and 0.66 ± 0.09 μM/s, respectively. For the forward reaction, the CfDHOase exhibited a *K*_*m*_ and *k*_*cat*_ of 0.846 ± 0.017 mM and 39.2 ± 6.4 per min, respectively, while for the reverse reaction the exhibited *K*_*m*_ and *k*_*cat*_ were 25.85 ± 2.67 μM and 258.6 ± 28.5 per min, respectively [28]. *Crithidia luciliae* dihydroorotase (ClDHOase) exhibited a *K*_*m*_ of 0.7 mM for dihydroorotate in the degradative direction. Forward reaction catalyzed by the DHOase in *L. donovani* and *C. fasciculata* was always favored as signified by kinetic parameter, which is biologically significant. LdDHOase displayed pH optimum of 6.0 for forward reaction, while pH optimum of 8.0 was observed for the reverse reaction. Kaempferol and biotin sulfone were able to inhibit LdDHOase with a *K*_i_ of 151 μM and 55 μM, respectively. However, the parasite did not show significant growth inhibition, which substantiated the fact that the parasitic growth was compensated by salvage pathway. Also, an increased expression of salvage pathway enzymes was observed when the *de novo* supply of pyrimidines was blocked. Thus, the inhibition of both pathways is necessary to retard the growth of the parasite [29].

### Dihydroorotate dehydrogenase (DHODH)

Dihydroorotate dehydrogenase (DHODH) is the fourth enzyme of the *de novo* pyrimidine biosynthesis pathway which has been extensively studied in trypanosomatids using a combination of genetic, biochemical and structural studies. DHODH catalyzes the oxidation of L-dihydroorotate to orotate, a redox reaction, the only one to do so in the pathway. DHODH in trypanosomatids has emerged as a crucial enzyme of the pyrimidine pathway, as the null mutants of TcDHODH and knockdown strains of TbDHODH displayed retarded growth phenotypes, confirming the crucial presence of DHODH in the pathway. However, the DHODH null background in *T. cruzi* could not be rescued by the supplementation of uridine, cytidine and thymidine. The non-reversal of the functional loss of DHODH by the pyrimidine supplemention was attributed to additional fumarate reductase activity of TcDHODH, which maintains the redox balance of the parasite [30]. TcDHODH was presumed to be a fumarate reductase owing to its close resemblance with *S. cerevisiae* type 1A DHODH, which was later confirmed by experimental evidence. The fumarate reductase activity of TcDHODH leads to the formation of succinate, explaining the observation of succinate overproduction by *T. cruzi* [31]. Cytosolic TcDHODH uses fumarate as an electron acceptor, while mitochondrial inner membrane bound mammalian DHODH uses ubiquinone as an electron acceptor; thus TcDHODH is not only crucial for pyrimidine metabolism but is also involved in the redox metabolism of the parasite. Abrogating the levels of DHODH in *T. brucei* resulted in retarded growth phenotype observed only in pyrimidine free media, signifying better salvaging capability of the bloodstream forms of *T. brucei* [32].

Extensive biochemical characterization of DHODH across trypanosomatid species has shed light on the reaction mechanism of the DHODH enzyme. DHODH has been biochemically characterized in *T. cruzi*, *T. brucei*, *L. major*, *L. mexicana*, *C. fasciculata* and *C. luciliae.* Kinetic parameters for TcDHODH catalyzed reaction were determined for both substrates dihydroorotate (*K*_*m*_ = 8.6 ± 2.6 μM and *V*_*max*_ = 4.1 ± 0.7 μM/s) and fumarate (*K*_*m*_ = 120 ± 9 μM and *V*_*max*_ = 6.71 ± 0.15 μM/s) [33]. Although three isoforms of TcDHODH exists, they all displayed the kinetic parameters in the same range. The three TcDHODH isoforms exhibited same activity in the pH range of 7.0–9.0 and were competitively inhibited by orotate. They displayed a higher *V*_*max*_ at 37 °C than at 25 °C, which is biologically significant, highlights its survival in the mammalian cells at physiological pH [34, 35]. TbDHODH catalyzed reaction proceeded with *K*_*m*_ values of 14 μM and 18 μM for dihydroorotate and fumarate, respectively (*k*_*cat*_^app^: 8.5/s), and the pH optima was estimated to be 7.8. LmDHODH catalyzes the reaction in a Ping Pong Bi-Bi manner and the estimated *K*_*m*_ values for dihydroorotate and fumarate were 90 ± 10 μM and 418 ± 67 μM, respectively, and the reaction proceeded with a *V*_*max*_ of 11 μmol/min. LmxDHODH revealed a *K*_*m*_ of 2.3 ± 0.04 μM and 11.8 ± 4.9 μM and a *V*_*max*_ of 55 nmol/h/mg and 30 nmol/h/mg for L-DHO in the promastigote and amastigote stages, respectively. The pH optima of *L. mexicana* dihydroorotate dehydrogenase (LmxDHODH) for the promastigote and amastigote stages was determined to be 7.0. CfDHODH yielded a *K*_*m*_ value of 1.3 mM for DHO. *Crithidia fasciculata* dihydroorotate dehydrogenase (CfDHODH) was subjected to substrate and product inhibition [36]; DHODH from *C. luciliae* (ClDHODH) exhibited a *K*_*m*_ of 5.8 μM with dihydroorotate as a substrate. DHODH in the trypansomatids are localized in the cytosol rather than in the inner membrane of mitochondria as in the mammalian cells. Thus, the use of site specific inhibitors for DHODH could bring out interesting results.

The activity of DHODH across the trypanosomatids has been inhibited by many potential inhibitors. Brequinar and leflunomide are effective inhibitors of human DHODH (a type II DHODH), targeting quinone binding sites which are absent in TcDHODH thus urging the need to design specific inhibitors of TcDHODH. An inhibitor specifically targeting TcDHODH need to be designed as it is visualized as an effective drug target in the *T. cruzi*, demonstrated by defective growth phenotypes exhibited by TcDHODH null mutants [33]. Orotate reportedly inhibits DHODH in a competitive manner across many species, an observation also reflected in the case of TcDHODH, where the inhibition was observed in the presence of substrates dihydroorotate and fumarate. Among the various screened algal extracts, extracts from two brown algae, *Fucus evanescens* and *Pelvetia babingtonii*, inhibited the TcDHODH in a non-competitive manner with a significant inhibition of 59% and 58% (*K*_i_ 35.3 ± 5.9 and 10.3 ± 4.4 μg/ml), respectively. Furthermore a decrease in the infectivity of *T. cruzi* parasites was observed in the presence of aforementioned algal extracts [37]. Inhibitors specific to EcDHODH were able to inhibit TbDHODH. 3, 4-dihydroxybenzoate inhibited TbDHODH in a competitive manner, with a *K*_i_ of 58 μM, while another inhibitor 3, 5-dihydroxybenzoate inhibited TbDHODH in a non-competitive manner, with a *K*_i_ of 200 μM [32]. Also the enzyme was inhibited in a competitive manner by pyrimidine analogs 5-methylorotate and 5-aminoorotate, with a *K*_i_ of 8.8 μM and 57 μM, respectively. It was also demonstrated that the enzyme was not coupled to the electron respiratory chain as the enzymatic activity was not affected on treatment with cyanide, antimycin A, TTFA or amytal. The enzyme exhibits a similar pH optimum for both stages of the parasites; however, different stages of *Trypanosoma* and *Crithidia* exhibit differing pH optima values. The activity of the dihydroorotate dehydrogenase of *L. mexicana* was mainly cytosolic. In a general consensus it can be said that trypanosomatid DHODHs are sensitive to pyrimidine analogs which can exert inhibitory effects on them.

Crystal structures of DHODHs in trypanosomatids have been reported in *T. cruzi*, *T. brucei* and *L. major*. DHODHs from *T. cruzi*, *T. brucei* and *L. major* are all dimers composed of a α/β barrel with FMN as a prosthetic group and follow a Bi-Bi ping pong mechanism of action, as shown by crystallographic and other structural studies. The crystal structure of TcDHODH diffracted at 2.2 Å proving it a homodimer, which was also supported by dynamic light scattering and analytical gel filtration chromatography studies. TcDHODH belongs to class 1A of DHODHs, which are cytosolic and harbor a cysteine residue in their active site, and uses fumarate as an electron acceptor. Site directed mutagenesis studies have shown that dimerization of DHODH is important for its activity. This property could be exploited to design ligands which could disrupt the dimerization and thus the activity. The crystal structure of TcDHODH has been reported in apo form as well as in bound form with orotate. Crystal structure of TcDHODH also revealed that substrates dihydroorotate, orotate, fumarate and succinate were all bound to the same site [38]. The monomeric TcDHODH comprises of 16β strands, 9 helices and 2 3_10_ helices folding into characteristic α/β fold. The TcDHODH shares only 28% amino acid sequence similarity with the mammalian counterpart. The crystal structure of TcDHODH would help in delineating the key differences between mammalian and TcDHODH, which could further be exploited for rational drug designing [39, 40]. A ping-pong Bi-Bi mechanism is followed by TcDHODH, where dihydroorotate binds first to the enzyme followed by fumarate, a mechanism revealed by the use of barbiturate (BA) a known dead end inhibitor of DHODH. In another study reaction mechanism of TcDHODH was studied by employing hybrid Quantum Mechanical/Molecular Mechanical (QM/MM) Molecular Dynamics (MD) simulations [41]. FMN was identified as a prosthetic group of TcDHODH by TLC analysis [31]. Crystal structure of TbDHODH was solved at a resolution of 1.95 Å and was shown to be a dimer composed of a α/β barrel with FMN as a prosthetic group situated at the C-terminus end. The dimeric nature of TbDHODH was also supported by analytical centrifugation studies. Analytical gel filtration chromatography showed that LmDHODH exists in the form of a homodimer [42]. The crystal structure of LmDHODH has been reported in apo form as well as in bound form with orotate and fumarate. Both orotate and fumarate were bound to the same active site supporting the Ping Pong Bi-Bi mechanism of catalysis. The dimeric nature of the LmDHODH was also supported by crystallographic evidence. The structure of LmDHODH revealed the presence of characteristic α/β barrel fold belonging to class 1A DHODH. The dimeric structure of DHODH closely resembles the structures of TbDHODH, TcDHODH and LdDHODH. Cys131 has been designated as the catalytic residue of LmDHODH [43].

### Uridine monophosphate synthetase (UMPS)

The specialized peroxisomes of trypanosomatids referred to as glycosomes also harbor various enzymes of the pyrimidine metabolic pathway. The bifunctional uridine monophosphate synthetase (UMPS) enzyme (comprised of orotate phosphoribosyltransferase (OPRT) and orotidine 5′-monophosphate decarboxylase (OMPDC)); catalyzing the last step of the *de novo* biosynthetic pathway, is localized in the glycosomes in case of trypanosomatids [6]. A C terminal glycosome targeting signal (SKL) present in the bifunctional UMPS enzyme localizes the enzyme to the glycosome [44]. An in silico analysis done in three trypanosomatids (*L. major*, *T. cruzi* and *T. brucei*) showed the presence of PTS1 (peroxisomal targeting sequence) located at the C terminal, targeting the protein to glycosomes which further supports the experimental evidences of their association with glycosomes [45]. Glycosomal association of UMPS has been experimentally demonstrated in case of TcOPRT, LmxOPRT and ClOPRT [46]. Preliminary work done on OPRTase and ODCase of *C. luciliae* showed that the enzyme was associated with glycosomes, an organelle unique to kinetoplastids using sucrose density gradient centrifugation. It was also shown that the two enzymes existed associated with each other in the form of a complex, which was one of the preliminary reports in the kinetoplastids. The OPRTase and ODCase complex could be solubilized in the presence of Triton X-100. The complex formation and their association with the glycosomes were thought to support the channeling of the product of one enzyme and substrate of the second.

Trypanosomatid UMPS are different from their host counterparts as they are localized in the glycosomes, and also the domains representing OPRT and OMPDC are reversed in the host counterpart. In the mammalian UMPS the OPRT is at the N terminus, while OMPDC is at the C terminus. These distinctions can be exploited for the specific targeting of trypanosomatid UMPS enzymes.

UMPS null mutants in *T. brucei* and *L. donovani* have shed light on their specific involvement in the growth and infectivity of the parasite. TbUMPS and LdUMPS null mutants displayed retarded growth phenotypes [47]. During earlier studies, it was not clear whether the *de novo* synthesis of UMP or its uptake from the extracellular environment was crucial. This was possibly because the probable uracil transporter in the procyclic *T. brucei* TbU1 was not found in the bloodstream forms of *T. brucei*; instead, another transporter TbU3 was present in the bloodstream forms. To assess the probable route for UMP synthesis, null mutants (*PYR* 6-5^-/-^) of *T. brucei* UMP synthase (TbUMPS) lacking the final steps of the *de novo* synthesis of pyrimidines were assessed for their abilities to survive in the pyrimidine free medium. The null mutants grew poorly in the pyrimidine free medium but were able to recover in the in vivo model, suggesting efficient transport of uracil which was able to circumvent the phenotype caused by the null mutants. However, the infectivity of the (*PYR* 6-5^-/-^) null mutants in *T. brucei* remained unaltered, as they overexpressed uridine phosphorylase and TbU3 transporter to compensate the null background [48]. A similar observation was noted in the case of *L. donovani* [47]. Uridine (100 μM) and cytidine (1 mM) supplementation could restore the normal growth phenotype in *T. brucei* (*PYR* 6–5^−/−^) null mutants, while 2′-deoxyuridine had almost no effect. However, uracil supplementation was more effective (GC_50_ value in micromolar range) then uridine supplementation in restoring the growth of *T. brucei* null mutants, possibly because of the presence of high affinity uracil transporter in *T. brucei*. The transport kinetics of uracil followed Michaelis Menten kinetics, with upregulation observed in the case of UMPS null mutants, while no upregulation for uridine was observed in UMPS null mutants. Cytosine, cytidine, thymine and thymidine supplementation also failed to restore the growth of TbUMPS null mutants [49]. Due to hampered synthesis of pyrimidines in (*PYR* 6-5^-/-^) null mutants, the DNA integrity was compromised resulting in aberrant chromosomes which led to cell death. Also (*PYR* 6-5^-/-^) null mutants were unable to undergo normal cell division. Although the infectivity of (*PYR* 6-5^-/-^) null mutants was unaffected as displayed in in vivo studies, the null mutants were able to salvage out pyrimidines available from the host [48]. *Trypanosoma brucei* pyr5 and pyr6 null mutants exhibited normal phenotype with unaltered growth and infectivity.

In another study, UMPS null mutants were generated by Herpes simplex virus thymidine kinase replacement, which altered the phenotypes of the null mutants as thymidine kinase has substrate specificity for deoxypyrimidines. A new study was undertaken in which null mutants of *T. brucei* UMPS were generated by nutritional rescue strategy. The *T. brucei* null mutants were unable to induce any robust infection in mice model, suggesting the essentiality of UMPS in the *T. brucei* parasite; however, there were occasional reports of infected mice with UMPS null mutants. These findings strengthened the fact that *T. brucei* parasites are able to take up free pyrimidines from the host cells for their survival and growth and that a combinatorial therapy blocking both the routes of pyrimidine biosynthesis would work for African trypanosomiasis caused by *T. brucei* parasites [49].

UMPS has also been biochemically investigated in the *Trypanosoma*, *Leishmania* and *Crithidia*. TcOPRT substrates orotate and 5′-phosphoribosyl-α-l-pyrophosphate exhibited a *K*_*m*_ of 2 μM and 8 μM, respectively. Uracil had no inhibitory effect on TcOPRT, while 5 fluoroorotate strongly inhibited TcOPRT [50]. TcOPRT displayed a pH optimum of 9.0. LdOMPDC was biochemically characterized with a *k*_*cat*_/*K*_*m*_ value of 1.2 × 10^4^ while for LdOPRT the *k*_*cat*_/*K*_*m*_ was 9.4 × 10^4^ and 7.9 × 10^4^ for orotate and PRPP, respectively. The kinetic parameters and the rate of reactions were not altered when the combination of enzymes were used. For *L. mexicana* it was revealed that the activity of the enzyme in the amastigote form of the parasite was 40 times lower than in the promastigote form of the parasite. Also a lower pH optimum was observed in the promastigote stage for OPRTase/ODCase enzymes. The bifunctional enzymes OPRTase and ODCase of *C. luciliae* were also biochemically characterized; the apparent *K*_*m*_ for the orotate as a substrate was determined to be 10 μM for OPRTase, similarly apparent *K*_*m*_ for OMP was determined to be 7.5 μM for OMPDCase [51]. A prodrug called pyrazofurin which metabolizes to 5′-monophosphate derivative, a potent inhibitor of mammalian orotidine 5′ monophosphate decarboxylase (OMPDC), was also tested for its inhibitory effects on *de novo* pyrimidine pathway; however, the inhibition was mainly due to off target effects [52].

The crystal structure of LdUMPS has been reported in complex with UMP and it exists in the form of a tetramer, shown by size exclusion chromatography. The tetrameric structure of LdUMPS comprises of two dimers of OPRT and OMPDC; thus, it is a dimer of dimers. It was also shown that the binding of the product in the active site of the enzyme led to the oligomerization of the enzyme, substantiated by circular dichroism (CD) studies, which pointed out an increase in the helical content upon product (UMP) binding [47]. Absence of ligand resulted in the dimeric form of LdUMPS, shown by size exclusion chromatography. There are similar reports of ligand induced oligomerization of UMPS for other species, especially in *P. falciparum* UMPS. Asp81 and Lys84 form the catalytic residues of LdOMPDC and many other residues were found to be conserved.

### Uracil phosphoribosyl transferase (UPRT)

The intracellular amastigote form of the *T. cruzi* is devoid of the uracil phosphoribosyl transferase (UPRT) and uridine kinase (UKase) enzymatic activities [53].

The uniqueness of the uracil phsophoribosyl transferase (UPRT) enzyme of the salvage pathway lies in the fact that this enzyme is absent in the mammalian system, thus making it a viable target for the development of the therapeutic intervention for the treatment of the visceral leishmaniasis (VL). UPRT is a key enzyme of the pyrimidine salvage pathway as it catalyzes the formation of UMP from uracil. UMP is a central metabolite of the pyrimidine pathway which can be further converted to various other pyrimidine metabolites and nucleotides. Owing to its unique presence, the UPRT enzyme was characterized in *L. donovani*. The essentiality of the UPRT enzyme was verified by generating null mutants of *L. donovani* UPRT. Biochemical characterization of *L. donovani* UPRT (LdUPRT) revealed a *K*_*m*_ of 20 and 99 μM for the uracil and phosphoribosylpyrophosphate (PRPP) substrates, respectively, and a pH optimum between 7.5–8.5. The reaction catalyzed by LdUPRT proceeded with a *V*_*max*_ of 13.6 ± 1.4 μM/min. For the pyrimidine analogs 5-fluorouracil and 4-thiouracil, the estimated *K*_*m*_ values were 6 and 7 μM, respectively. LdUPRT exists in the form of a tetramer, as shown by size exclusion chromatography. The catalytic activity of LdUPRT was also shown to be dependent on the divalent cation like Mg^2+^. Substrate level inhibition of LdUPRT could be observed when uracil was taken up to a concentration 10 times of *K*_*m*_ value [54].

Since LdUPRT was also susceptible to substrate level inhibition by uracil analogs, these analogs were tested for their inhibitory effects on the growth of pyrimidine auxotrophs of *L. donovani* parasites. *Leishmania donovani* pyrimidine auxotrophs (cps and umps) were sensitive toward 4-thiouracil, with an EC_50_ of 82.3 μM and 67.3 μM, respectively; the wild type parasites and uprt null mutants remained unaffected in the presence of 4-thiouracil. Thus, it is shown biochemically that 5-fluorouracil was phosphoribosylated by UPRT and UMPS [54].

UPRTase was purified from *C. luciliae* and biochemically characterized. The enzyme was determined to be a dimer. The determined pH optimum for the ClUPRTase was between 8.5–9.0. The enzyme utilizing uracil and P-Rib-PP as a substrate proceeded with *K*_*m*_ of 37 and 95 μM, respectively. The ClUPRTase was shown to be inhibited by ATP but failed to display any stimulation by GTP [55].

### Deoxyuridine-triphosphatase (dUTPase)

Cellular pools of dTTP and dUTP are balanced by deoxyuridine-triphosphatase (dUTPase), a unique enzyme of the pyrimidine pathway by hydrolyzing dUTP to dUMP in an Mg^2+^-dependent manner thus ensuring DNA integrity. Lower cellular pools of dUTP will prevent misincorporation of uracil in DNA during replication and repair processes which otherwise may lead to the generation of fragmented DNA and cell death.

The essentiality of dUTPase in the trypanosomatids has been established by the null mutants of dUTPase in *T. brucei* and *L. major*, as they had reduced viability [56]. Further knockdown studies on dUTPase in *T. brucei* and *L. major* led to increased dUTP cellular levels, which resulted in DNA strand breaks due to misincorporation of uracil in the DNA which led to increased expression of glycosylase, as it is involved in base excision repair and enlarged nucleus. TbdUTPase depletion also affected cell cycle progression as dUTPase depleted cells were unable to cross G2/M phase of cell cycle in *T. brucei* because of DNA strand breaks, estimated by FACS analysis [57]. Proliferation arrest in *T. brucei* dUTPase null mutants could not be rescued by uracil, uridine and deoxyuridine supplementation [58]. Dual activity of *Leishmania* dUTPases was established, with cell free extracts of *Leishmania* in the presence of dUTP leading to the formation of dUDP as well as dUMP.

The reported *K*_*m*_ values for TcdUTPase and *L. major* dUTPase (LmdUTPase) were between 0.2–1.2 μM and the reaction generally proceeded with a slower rate (*k*_*cat*_: 2.8/s and 49/s, respectively). DMT-dU, a known inhibitor of *E. coli* dUTPase, failed to inhibit TcdUTPase and *Leishmania* dUTPase, owing to structural differences between the *E. coli* and parasitic dUTPase. Another compound, K-L-imido-dUDP, turned out to be an effective inhibitor of TcdUTPase with a *K*_i_ of 0.24 μM [59]. Also some uracil conjugates were screened as putative inhibitors using in silico approaches for TcdUTPase [60]. TbdUTPase was also biochemically characterized and the *K*_*m*_ and *k*_*cat*_ values for dUTP were 1.79 ± 0.3 μM and 9.7/s, respectively. Biochemical characterization of LmdUTPase revealed that the enzyme catalyzed the reaction with a low value for dUTP (*K*_*m*_: 2.11 μM). The enzyme followed Michaelis-Menten kinetics and also catalyzed the hydrolysis of dUDP (*K*_*m*_: 62 μM, *V*_*max*_: 99 units/mg). LmdUTPase has a high affinity towards dUTP compared to dUDP, evident from the lower *K*_*m*_ value for dUTP. Kinetic estimation of LmdUTPase also revealed the dependence of the enzyme on the presence of Mg^2+^, as the enzyme lost its activity when the metal ion was replaced with Ca^2+^ or Cu^2+^. The LmdUTPase was also competitively inhibited by DMT-dU (*K*_i_ > 1000 μM). While α-β-imido-dUTP turned out to be a potent inhibitor of LmdUTPase (*K*_i_: 0.89 μM) (42). The binding of 2'-dUMP to dUTPase was also estimated by isothermal titration calorimetry (ITC) in case of LmdUTPase [61].

Crystal structure for *T. cruzi* dUTPase (TcdUTPase) has been reported in apo form as well as in dUDP bound form. TcdUTPase exists in the form of a dimer which has been shown by size exclusion chromatography and crosslinking studies [59]. Dimeric dUTPases are present in *T. cruzi*, *T. brucei* and *L. major*. The dimeric trypanosomatid dUTPases are structurally distinct from trimeric mammalian dUTPases as they lack the characteristic motifs present in the mammalian dUTPases. The diversified structure of TcdUTPases opens up a wide range of possibilities for specific drug design [62]. The ability of TcdUTPase to hydrolyze dUTP, as well as dUDP, distinguishes it from the trimeric dUTPases. The catalytic role of Asp80 residue of TcdUTPase was demonstrated by an Asp80Ala mutation in TcdUTPase, which altered its capability to distinguish between dUTP and dUMP while retaining the proper active site orientation, where dUMP and dUTP exhibited a non-cooperative mode of binding to the Asp80Ala mutant. This study showed the catalytic role of Asp80 residue of TcdUTPase, helpful in the rational drug designing process [63]. Crystal structure of *L. major* dUTPase (LmdUTPase), a crucial enzyme of the pyrimidine salvage pathway has been reported with product (UMP) bound to it. The dUTPase from trypanosomatids has the remarkable ability to hydrolyze dUTP as well as dUDP. The LmdUTPase exists in the form of a dimer like all kinetoplastids, shown by gel filtration chromatography experiments [64]. The rigid domains of the protein mediated the formation of dimers. LmdUTPase is a metal containing enzyme with Mg^2+^ present in the enzyme structure [65]. TbdUTPase was localized in the nucleus as evident from immunofluorescence studies.

### Uridine phosphorylase (UPase)

Uridine phosphorylase (UPase) is a crucial enzyme of pyrimidine salvage pathway which catalyzes the formation of uracil from uridine and 2′-deoxyuridine in a reversible reaction. *Trypanosoma cruzi* and *T. brucei* uridine phosphorylase exists in the form of a homodimer, estimated by gel filtration chromatography. Crystal structure of uridine phosphorylase (UPase) has been reported from *T. brucei* which exists in the form of a dimer. Ca^2+^ was present in the active site of TbUPase which may play a role in the structural stability of the enzyme.

Kinetic characterization of *T. cruzi* uridine phosphorylase (TcUPase) revealed that with uridine and 2′-deoxyuridine as substrates it had a *K*_*m*_ of 21 ± 2 μM (*k*_*cat*_: 12 ± 1/s) and 32 ± 4 μM (*k*_*cat*_: 15 ± 1/s), respectively [66]. TbUPase displayed maximum activity with uridine as a substrate at a pH of 7.5. Depletion of TbUPase had no effect on the growth of *T. brucei* parasites and they exhibited normal growth, suggesting this enzyme is not essential [67]. Uridine phosphorylase (UPase) of *C. luciliae* catalyzed the reaction with a *K*_*m*_ of 2.0 and 2.2 mM for the catabolic direction, while it had a *K*_*m*_ of 7.5 mM for uracil in the anabolic direction [68].

### Cytidine deaminase (CDA)

Cytidine deaminase (CDA) from *T. cruzi* was biochemically characterized and various kinetic parameters were determined. *Trypanosoma cruzi* cytidine deaminase (TcCDA) mediated reaction proceeded with a *K*_*m*_ of 1.9–1.8 mM for cytidine and deoxycytidine as a substrate [69]. The activity of cytidine deaminase (CDA) yields the formation of uridine and deoxyuridine, respectively. pH optimum for cytidine deaminase from *C. fasciculata* was 6.5–8.5. The cytidine deaminase from *C. fasciculata* (CfCDA) displayed lower affinity for cytidine (3–2.8 mM) over deoxycytidine (0.1–0.09 mM) [69]. Depletion of cytidine deaminase (CDA) also led to a decrease in the growth of *T. brucei* parasites. In case of CDA null mutants, exogenous deoxyuridine and deoxythymidine were able to rescue the growth defect [70].

### CTP synthetase (CTPS)

CTP synthetase (CTPS) is a glutamine amidotransferase which carries out the synthesis of CTP and has been extensively characterized in *T. brucei*. *Trypanosoma brucei* has lower CTP pools because of slower synthesis by *T. brucei* CTP synthetase (TbCTPS) making this a vulnerable step [21, 71]. The slower synthesis of CTP by TbCTPS, and poor cytidine salvage, makes TbCTPS a good drug target. The CTP pool of *T. brucei* was unlabelled when grown in the presence of tritiated cytidine and cytosine, signifying their inabiliy to salvage cytidine or cytosine [21]. In a similar manner, null mutants of *T. brucei* CTP synthetase (CTPS) were not able to assimilate extracellular cytosine [21]. 3-bromoacivicin binds to the glutaminase domain of TbCTPS, inhibiting it by covalent modification, but failed to reduce the viability of trypanosomes, thus urging the requirement to test more potent inhibitors [72]. Characterization of the glutamine dependent TbCTPS has been reported previously [73]. Another study reported the inhibition of TbCTPS by GTP and purine analogs. TbCTPS was also shown to be inhibited in vitro and in vivo by glutamine analogs 6-diazo-5-oxo-L-norleucine (DON) and a-amino-3-chloro-4,5-dihydro-5-isoxazoleacetic acid (acivicin) [21]. A modified version of acivicin a-amino-3-bromo-4, 5-dihydroisoxazol-5-yl acetic acid (3-bromoacivicin) was synthesized and found to be three times more potent than its parent compound [72]. Acivicin is a well known drug which can penetrate the blood brain barrier and can prove to be a good therapeutic for the treatment of African sleeping sickness. Acivicin inhibited TbCTPS with a *K*_i_ of 2.3 μM. The ability of TbCTPS to utilize glutamine and ammonia as substrate was also established [73, 74]. The estimated *K*_*m*_ and *k*_*cat*_ values for ammonia as a substrate for TbCTPS were 2.2 mM and 1.6/s, respectively. TbCTPS displayed lesser activity as compared to EcCTPS. TbCTPS was reported to be activated by GTP at [GTP] < 0.2 mM and inhibited by GTP at [GTP] > 0.2 mM [74]. The lower CTP pools in *T. brucei* were also attributed to the higher *K*_*m*_ (0.16 mM) value exhibited by TbCTPS for UTP when compared to the mammalian CTPS (*K*_*m*_: 0.07 mM). TbCTPS exists in the form of a tetramer, shown by GEMMA technique (gas-phase electrophoretic mobility macromolecule/nanoparticle analysis) [73]. Thus the *de novo* synthesis of CTP and the absence of CTP salvaging in the *T. brucei* can be utilized for the development of CTP inhibitor based therapeutics as host has the ability to salvage cytidine and cytosine [21].

### Thymidine kinase (TK)

Thymidine kinase (TK) is another significant enzyme of the pyrimidine salvage pathway which phosphorylates thymidine to form TTP. Mammalian cells possess two variants of thymidine kinase, TK1 (cytosolic) and TK2 (mitochondrial), but only TK1 is present in trypanosomatids. A monomeric thymidine kinase was found to exist in *T. brucei*, as estimated by gel filtration chromatography; however, a pseudodimer is also present because of two domains. TbTK was comprised of two domains of which only domain 2 (C terminal domain) was found to be kinetically active [75]. A single domain is present in LmTK, in contrast to two domains of TbTk. The crystal structure of LmTK has been reported with substrate dThd bound to it and it was shown to be a tetramer by analytical centrifugation technique [76].

Although *T. brucei* possess a functional *de novo* pathway and thus have minimum reliance on the salvage pathway, the depletion of TK, a salvage pathway enzyme, led to a decrease in the growth and infectivity of the *T. brucei* parasites. TbTK is responsible for the formation of the dUMP and dTMP. The retarded growth phenotype was also perhaps due to a lack of dCMP deaminase enzyme, which provided an alternative route for the formation of dUMP. Ectopic expression of dCMP deaminase was able to rescue the retarded growth phenotype observed because of RNAi mediated knockdown of TbTK. However, the growth defect of TK depleted cells could not be compensated by exogenous supplementation of pyrimidines. Also, the levels of deoxyuridine and deoxythymidine, the substrates of TK, were found to be accumulated in the TK depleted *T. brucei*; however, the CMP and UMP levels remained unaffected signifying that *de novo* pathway was able to make up the desired cellular pool of these pyrimidines [70]. Furthermore, null mutants of LmTK exhibited short flagellum as compared to the wild type parasites. A significant reduction in infectivity was also observed in the LmTK null mutants [77].

Thymidine kinase (TK) leads to the formation of thymidine mono phosphate (dTMP), which is further phosphorylated to form dTTP, a substrate of DNA polymerase. *Leishmania major* thymidine kinase (LmTK) has been demonstrated to be crucial in maintaining the infectivity of the parasite. LmTK was biochemically characterized and its structure was determined by crystallography. LmTK does not use purine as substrates unlike TbTK, also LmTK was found to be inhibited by dUTP. The enzyme catalyzed the reaction with a *K*_*m*_ and *k*_*cat*_ of 1.1 μM and 2.62/s for deoxythymidine, respectively, and exhibits cooperative behavior. Thymidine kinase in the *Leishmania* species have a 100 amino acid C terminal extension and belongs to Type II category, based on amino acid sequence comparison. Thymidine kinase in the *L. major* is localized in the cytosol, which was determined by immunoelectron microscopy. A positive cooperativity for ATP was exhibited by LmTK, with a Hill coefficient of 1.6. The observed *K*_*m*_ value for dUrd was 1462 μM, thus the enzyme had a lower affinity for dUrd. dUTP was found to inhibit LmTK with a IC_50_ value of 187 μM [76]. Also, a higher expression of thymidine kinase has been reported in the amastigote form of *L. major* and it was inferred that a higher expression probably suggests a possible shift towards the salvage pathway in the amastigote stage.

### Nucleoside hydrolase

Nucleoside hydrolase from *C. fasciculata* was biochemically characterized and exhibited high affinity for purines over pyrimidines. The oligomeric status of the enzyme was determined to be a tetramer from gel filtration studies [78]. Uridine kinase (UKase) activity is absent in trypanosomatids [50].

## Pyrimidine transport in trypanosomatids

**Fig. 1:**
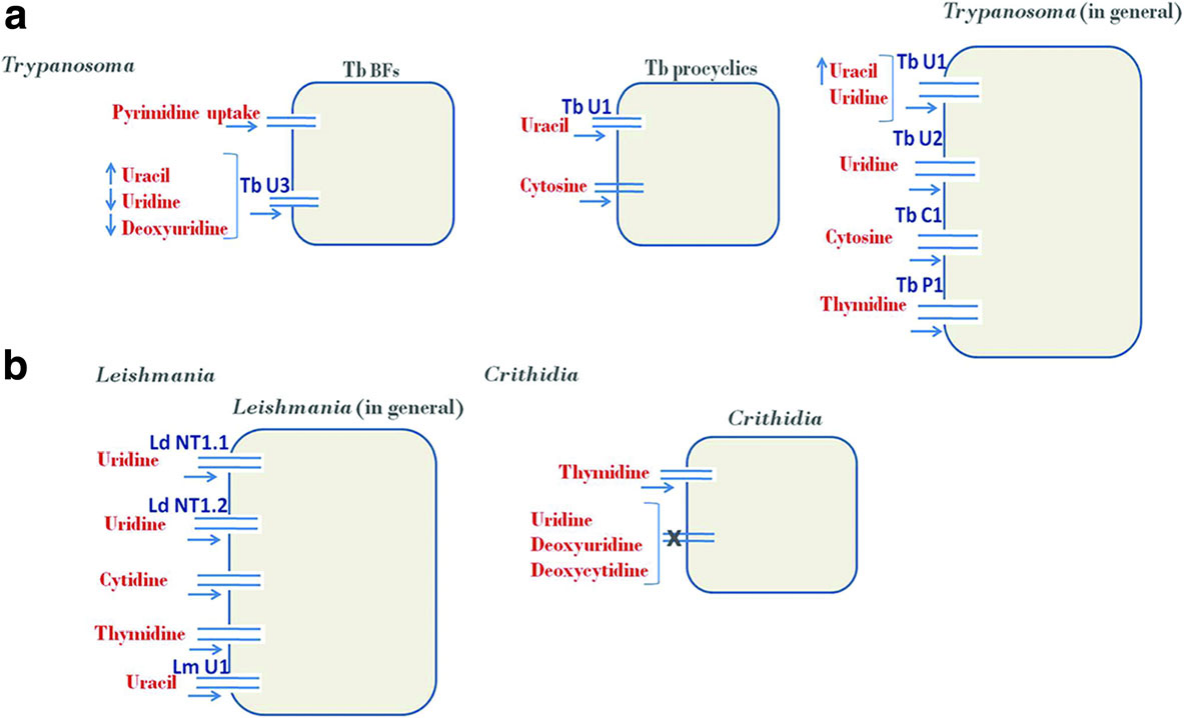
Schematic representation of various transporters involved in the pyrimidine transport in trypanosomatids. **a** Pyrimidine transport in *Trypanosoma*. **b** Pyrimidine transport in *Leishmania* and *Crithidia. Abbreviation*: TbBFs, *Trypansosoma* blood stream forms

Uracil uptake in the *T. brucei* procyclic forms is mediated by a high affinity uracil transporter U1 (a proton symporter) which uptakes uracil following Michaelis-Menten kinetics with a *K*_*m*_ and *V*_*max*_ of 0.46 ± 0.09 μM and 0.65 ± 0.08 pmol/s, respectively (Fig. 1a). Two uridine transporters U1 and U2 with moderate affinity have been implicated in *T. brucei* but primarily U1 is a uracil carrier. A variation in the salvaging capacity of *T. brucei* procyclic and bloodstream forms has been reported where procyclic and bloodstream forms display distinct transporters especially in the case of uridine and uracil transport. In the procyclic forms of *T. brucei* TbU1 is the uracil carrier, while in the bloodstream forms TbU3 is the uracil carrier. *Trypanosoma brucei* is equipped with three kinds of pyrimidine transporters (U1, U2 and C1) along with a uracil transporter (U3), which is active in the mammalian stage of infection. U3 exhibited higher affinity for uracil than for uridine and deoxyuridine [79].

Uptake of uracil was preferred, even in the presence of uracil analogues such as 5-chlorouracil, 3-deazauracil, and 2-thiouracil, which demonstrated the high affinity and specificity of the uracil transporter U1. However, uracil uptake was inhibited in the presence of 5-fluorouracil (*K*_i_ = 3.2 ± 0.4 μM) and uridine (*K*_***i***_ = 48 ± 15 μM) [80]. Furthemore, in another study uridine uptake by same uracil transporter (U1) was demonstrated with a *K*_*m*_ of 33 ± 5 μM, although a high affinity uridine transporter (U2) with a *K*_*m*_ of 4.1 ± 2.1 μM and a *V*_*max*_ of 0.019 ± 0.0086 pmol/s has also been described [81] and is inhibited by thymidine (*K*_i_: 0.38 ± 0.07 μM) and cytidine (*K*_i_: 0.041 ± 0.023 μM). Cytosine uptake was demonstrated by high affinity C1 transporter (*K*_*m*_: 0.048 ± 0.009 μM) and its uptake was inhibited by cytidine (*K*_i_: 0.42 ± 0.16 μM) and uracil (*K*_i_: 0.36 ± 0.06 μM) [81]. Availability of thymine, thymidine and cytidine in *T. brucei* seems to be made out by synthesis from UMP but not by specific transporters [81]. However, another report suggests that uptake of thymidine is done inefficiently by a P1 type nucleoside transporter.

A high affinity uracil transporter (TbU3) in the bloodstream forms of *T. brucei* was also characterized. The TbU3 transporter had high affinity towards uracil and a lower affinity towards uridine and 2′-deoxyuridine. However, 5-fluorouracil was found to be good substrate for TbU3 transporter and exhibited trypanocidal activity. The uracil uptake in the bloodstream *T. brucei* forms proceeded at a *K*_*m*_ and *V*_*max*_ of 0.54 μM and 0.14 ± 0.01 μM/s. *V*_*max*_ values imply that a lower uptake rate of uracil is being observed in the bloodstream forms then in the procyclic forms of *T. brucei*. *Trypanosoma brucei* bloodstream forms were also found to be inefficient in the uptake of cytidine [79]. Bloodstream forms of *T. brucei* rely solely on CTP synthase for the cytidine pool, as they lack cytidine transport activity, while the same is being observed in the procyclics as they possess a cytosine transporter [21]*.* All these studies have pointed towards the fact that pyrimidine salvage pathway primarily relies on the transport of uracil. These studies also highlight that different morphological forms of *T. brucei* express different pyrimidine transporters, but how this differential expression of transporters in *T. brucei* morphological forms is regulated remains to be elucidated. Among the various pyrimidine analogs used, 5-fluorouracil was found to be more effective on *T. brucei.* The conversion of 5-FOA to 5-UMP was found to be more efficient than the conversion of 5-FU by phosphoribosylation.

Purine transport has been dealt in great details in *L. donovani* but the importance of pyrimidines has not been investigated in detail. Few studies have indicated the presence of high affinity uracil transporters in the *Leishmania* parasite [80, 82]. Nucleoside transporters have been identified in *L. donovani*, which enables the parasite to take up uridine, cytidine and thymidine. Uridine at a concentration of 1 μM was transported in linear fashion by LdNT1.1 (5.6 ± 1.8 μM) and LdNT 1.2 (40 ± 11 μM), respectively. The two permeases (LdNT1.1 and LdNT1.2) have a difference of only six amino acid residues, which may account for their differing affinities for uridine (Fig. 1b). Uridine uptake was inhibited in a non-competitive manner by carbonyl cyanide m-chlorophenylhydrazone (CCCP), a proton gradient uncoupler [82]. *Leishmania donovani* also had the limited ability to salvage thymidine and orotic acid [83]. The presence of pyrimidine transporters have also been demonstrated by Vasudevan et al. [84].

LmU1, a *L. major* transporter, has been shown to be responsible for the uptake of uracil. The transporter mediated the uptake of uracil obeying the Michelis-Menten kinetics with a *K*_*m*_ and *V*_*max*_ of 0.32 ± 0.07 μM and 0.68 ± 0.15 pmol/s, respectively. The LmU1 transporter displayed high specificity as no uptake of other pyrimidines such as cytosine or thymine was reported. 5-fluorouracil also displayed high affinity for the LmU1 transporter. Various uracil analogs, such as 5-fluorouracil, 5-chlorouracil, 5-carboxyuracil and 5-methyluracil, were tested for deducing the mechanism of uracil transport by LmU1; it appears that LmU1 forms hydrogen bonds with the keto groups of uracil, thus mediating its uptake [12].

To gain more insights into the pyrimidine metabolism and the transport activities in *L. infantum*, a pyrimidine analog 5-Fluorouracil (5-FU) was employed and *L. infantum* mutants resistant to the antimetabolite 5-FU were analyzed. It was identified that the mutants had point mutations in uracil phosphoribosyl transferase (UPRT), uridine phosphorylase (UP) and thymidine kinase (TK), which was also confirmed by complementation studies. Also, one mutant had a deficiency in uracil and 5-Fluorouracil uptake. The 5-FU resistant mutants were generated by gradually increasing the concentrations of 5FU over several passages and the mutants obtained were genetically analyzed to identify any modifications. An increase in the copy number of DHFR-TS gene was observed in few mutants. Single nucleotide polymorphism (SNP) analysis in the 5-FU mutant *L. infantum* lines revealed point mutations in *UPRT*, *UP* and *TK* genes, which encode key enzymes of the pyrimidine salvage pathway. The sensitivity of the mutants was restored when they were transfected with the wild versions of the above mentioned enzymes, thus confirming that the resistant phenotype was due to the point mutations in the mentioned enzymes of the pyrimidine pathway. Uracil and 5-FU transport was also found to be defective in these mutants [85].

Nucleoside transport activities in *C. luciliae* were assessed by a rapid sampling technique, which revealed the presence of two nucleoside transporters, one of which transported thymidine along with purine nucleosides. The other nucleoside transporter was specific to the purine nucleosides only. Thymidine transport exhibited a 50% lesser rate compared to the transport of purine nucleosides. No uptake of uridine, deoxyuridine and deoxycytidine was demonstrated by *C. luciliae* [86].

## Conclusions

**Fig. 2:**
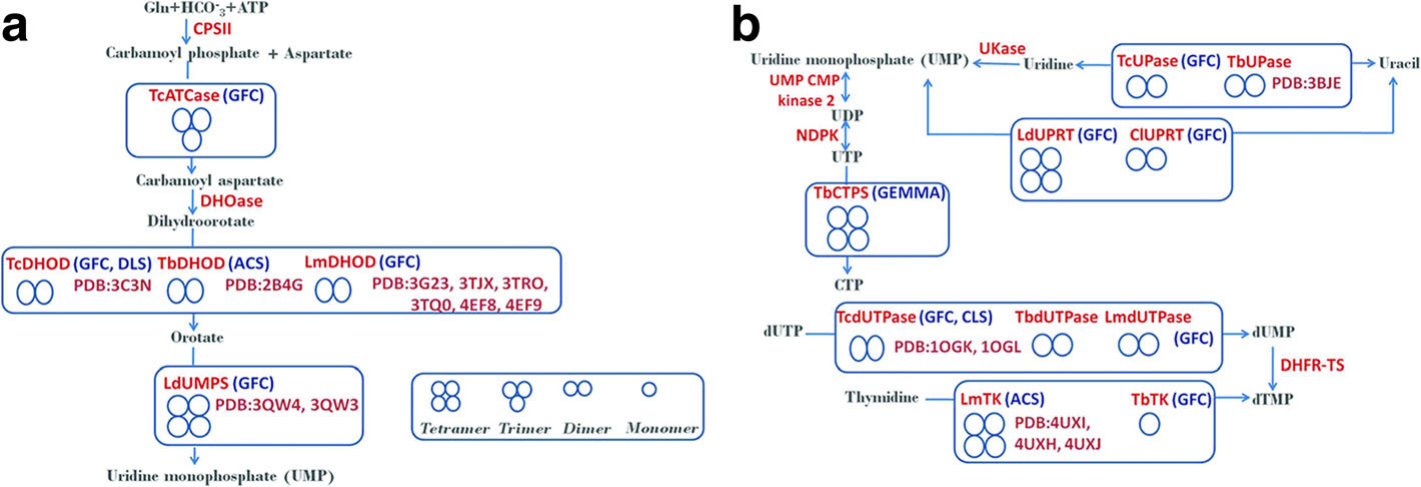
Schematic representation of the oligomeric states of the various enzymes involved in the pyrimidine metabolism of the trypanosomatids. **a** Enzymes involved in the *de novo* pyrimidine biosynthesis pathway. **b** Enzymes involved in salvage pathway for pyrimidines. The bracketed abbreviations are depicting the techniques employed for the estimation of oligomeric state of an enzyme. PDB ID is also mentioned if the crytstal structure of the given enzyme has been solved. *Abbreviations*: CPSII, carbamoyl-phosphate synthase; ATCase, aspartate carbamoyl transferase; DHOase, dihydroorotase; DHODH, dihydroorotate dehydrogenase; UMPS, uridine monophosphate synthetase; UKase, uridine kinase; UPase, uridine phosphorylase; UPRT, uracil phospho ribosyl transferase; NDPK, nucleoside-diphosphate kinase; dUTPase, deoxyuridine-triphosphatase; DHFR-TS, dihydrofolate reductase-thymidylate synthase; TK, thymidine kinase. Tc, *Trypanosoma cruzi*; Tb, *Trypanosoma brucei*; Ld, *Leishmania donovani*; Lm, *Leishmania major*; Cl, *Crithidia luciliae*; GFC, gel filtration chromatography; DLS, dynamic light scattering; CLS, cross linking Studies; ACS, analytical centrifugation studies; GEMMA, gas phase electrophoretic mobility macromolecule/nanoparticle analysis

Understanding biochemistry of parasites is key for the development of novel drug candidates [87–90]. Enzymes of pyrimidine metabolism, because of their function, can be a potential target for drug development. A multitude of techniques involving gel filtration chromatography, crosslinking studies and dynamic light scattering, along with crystallographic evidences, have revealed the oligomeric states of a plethora of enzymes involved in the pyrimidine pathway across trypanosomatids (species of *Trypanosoma*, *Leishmania* and *Crithidia*). As the enzymatic homologs of this ancient pathway in trypanosomatids are conserved at the genetic and proteomic level, a similar degree of relatedness has been reflected at the oligomeric states of the enzymes (Fig. 2). This notion has been exemplified by the enzymes like dihydroorotate dehydrogenase (DHODH), as the enzymatic homologs of this enzyme like TcDHODH, TbDHODH and LmDHODH are all dimers while the salvage pathway enzymes like uridine phosphorylase (UPase) and dUTPase are dimers too. These similarities at the genetic and structural level may strengthen the evolutionary relatedness of the trypanosomatid species and may be exploited in therapeutic interventions. Displaying closedness in terms of genetic and proteomic level is altered by a significant finding in the *Trypansoma* species in the first three steps of the *de novo* pyrimidine pathway. It has been the general consensus that there are three independent enzymes catalyzing the first three steps of the *de novo* route of pyrimidine synthesis in trypanosomatids, supported by the presence of leader sequences and splice sites in their mRNA sequences. But *Trypanosoma* has proved to be an exception, as the first three enzymes of the *de novo* pyrimidine pathway form a multifunctional enzyme complex in a manner similar to the host cells, which has been supported by co-immunoprecipitation studies. *Leishmania* and *Crithidia* still catalyze the first three steps of the *de novo* pyrimidine pathway by the three independent enzymes, as shown by gel filtration chromatography experiments. On the contrary, however, many enzymatic homologs exhibited differing behavioral attributes in different trypanosomatid species, despite of the fact that they trace their evolutionary lineage from the same group, i.e. trypansomatids. This fact is exemplified in the case of aspartate transcarbamoylase (ATCase) enzymes from *T. cruzi* and *L. donovani* as PALA, a specific inhibitor of ATCase, failed to inhibit TcATCase but was a good inhibitor of LdATCase. While *Leishmania* poses a tough front, with efficient salvaging (transporters) at one end, the same is posed by the *Trypanosoma* in the first three steps of the *de novo* pathway by the formation of the multifunctional enzyme complex, rendering it untargetable. While in *Leishmania* and *Crithidia*, this aspect is lenient because the existence of independent enzymes in the first three steps makes it vulnerable at these steps. Bloodstream forms of *Trypanosoma* instill some hope, as they are weak in the salvaging of pyrimidines.

Various morphological forms of *Trypanosoma* seem to regulate the differential expression of various transporters responsible for salvaging pyrimidines from the outside environment. Although all pyrimidines including uracil, uridine, cytosine and thymidine are transported by *Tryapansoma*, there is a tight control at the transport of these metabolites by the different morphological forms of *Trypanosoma.* How this control is being exerted by the *Trypanosoma* remains to be deduced. Thus, few morphological forms of *Trypanosoma* which are defunct or weak in the salvaging of pyrimidines from the extracellular environment can be exploited to design interventions for their removal from the host. How evolutionary selective pressure has yielded the differential modulation of these transporters in *Trypanosoma* needs to be explained, and an understanding of this aspect may bring out fresh insights in the control of these parasites. *Leishmania* is also equipped with various transporters (uracil, uridine, cytidine and thymidine) to aid in its survival in the host via salvage pathway. The salvage route of pyrimidine synthesis is well supported by a variety of pyrimidine transporters. Thus, contrary to *Trypanosoma*, *Leishmania* pose tough competition regarding their removal from the host, because of strongly developed routes of pyrimidine synthesis (*de novo* and salvage). Some sort of morphological bias is also being presented by the *Leishmania* group of parasites, as exemplified in the case of *L. major* thymidine kinase (LmTK), a salvage pathway enzyme whose expression is higher in the amastigote stage, and *L. mexicana* UMPS, a *de novo* pathway enzyme whose activity is 40 times lower in the amastigote stage of the parasite, signifying a shift towards the salvage pathway in the amastigote stage of the parasite. Despite of deep exploration of the pyrimidine metabolism in the trypanosomatids, a lot of factors remain elusive. A better understanding of the pyrimidine transport activities, along with characterization of some salvage pathway enzymes in the parasites, would aid in a better understanding of the biological circuitry of these group of parasites. There is an urgent need to exploit the vast amount of information available in pyrimidine metabolism in the trypanosomatids, which will further aid in the development of effective cures for the treatment of the deadly diseases caused by trypansomatids.

## Abbreviations

ACS: Analytical centrifugation studies
ATCase: Aspartate carbamoyl transferase
Cf: *Crithidia fasciculata*
Cl: *Crithidia luciliae*
CLS: Cross linking studies
CPSII: Carbamoyl-phosphate synthase
DHFR-TS: Dihydrofolate reductase-thymidylate synthase
DHOase: Dihydroorotase
DHODH: Dihydroorotate dehydrogenase
DLS: Dynamic light scattering
dUTPase: Deoxyuridine-triphosphatase
GEMMA: Gas phase electrophoretic mobility macromolecule/nanoparticle analysis
GFC: Gel filtration chromatography
Ld: *Leishmania donovani*
Lm: *Leishmania major*
Lmx: *Leishmania mexicana*
NDPK: Nucleoside-diphosphate kinase
Tb: *Trypanosoma brucei*
TbBFs: *Trypansosoma* blood stream forms
Tc: *Trypanosoma cruzi*
TK: Thymidine kinase
Ukase: Uridine kinase
UMPS: Uridine monophosphate synthetase
UPase: Uridine phosphorylase
UPRT: Uracil phospho ribosyl transferase
